# Transarterial chemoembolization is ineffective for neuroendocrine tumors metastatic to the caudate lobe: a single institution review

**DOI:** 10.1186/s12957-015-0551-4

**Published:** 2015-05-01

**Authors:** Lawrence A Shirley, Megan McNally, Ravi Chokshi, Natalie Jones, Patrick Tassone, Gregory Guy, Hooman Khabiri, Carl Schmidt, Manisha Shah, Mark Bloomston

**Affiliations:** Division of Surgical Oncology, The Ohio State University Wexner Medical Center, N924 Doan Hall, 410 W. 10th Ave, Columbus, OH 43210 USA; Department of Surgery, Saint Luke’s Health System and University of Missouri-Kansas City, 100 NE St. Lukes Blvd, Lee’s Summit, MO 64086 USA; Division of Surgical Oncology, Rutgers University-New Jersey Medical School, 205 South Orange Avenue, G-1222, Newark, NJ 07103 USA; Department of Surgery, Riverside Methodist Hospital, 1654 Upham Dr., Columbus, OH 43210 USA; Department of Otolaryngology, Thomas Jefferson University Hospital, 925 Chestnut Street, 6th Floor, Philadelphia, PA 19107 USA; Department of Radiology, The Ohio State University Wexner Medical Center, Room 460, 395 W. 12th Ave, Columbus, OH 43210 USA; Division of Medical Oncology, The Ohio State University Wexner Medical Center, 4th Floor Starling Loving Hall, 320 W. 10th Ave, Columbus, OH 43210 USA

**Keywords:** Neuroendocrine tumors, Liver metastases, Locoregional therapy, Interventional radiology

## Abstract

**Background:**

Caudate lobe liver metastases occur commonly in patients with neuroendocrine tumors. It is unknown, however, how these lesions respond to regional therapy and how their presence impacts outcomes. We reviewed our experience treating these lesions using transarterial chemoembolization (TACE).

**Methods:**

We reviewed radiographic response to TACE in 86 patients with metastatic neuroendocrine tumors to the liver. We determined the impact of caudate lesions on outcomes in comparison to the cohort of patients without caudate lesions, as well as response of caudate lesions to TACE *versus* lesions elsewhere in the liver.

**Results:**

Caudate lesions were identified in 45 (52%) patients. All patients had disease in other liver segments. Only seven caudate lesions (12.3%) had a radiographic response to TACE, whereas 82% of lesions elsewhere in the liver demonstrated a response. The presence or absence of a caudate lesion did not impact the overall radiographic (82.2% *vs*. 82.9%), symptomatic (64.4% *vs*. 56.1%), or biochemical (97.6% *vs*. 88.9%) response to TACE (*P* > 0.1 for all). However, median overall survival was reduced in those presenting with caudate lesions (87.1 *vs*. 45.6 months, *P* = 0.031).

**Conclusions:**

Metastatic neuroendocrine tumors to the caudate lobe respond poorly to TACE. Symptomatic or threatening caudate lobe lesions should be considered for palliative resection in spite of additional inoperable liver metastases.

## Background

Neuroendocrine tumors (NET) are often aggressive but indolent malignancies, which clinically do not manifest for years, even in the presence of extensive metastatic disease. Liver metastases are the most common site of distant spread and are the primary threat to life [1]. Given the inherent resistance of neuroendocrine tumors to systemic chemotherapy and the predilection for liver metastases, liver-directed therapies such as transarterial chemoembolization (TACE) play an important role in treatment [2-4].

The caudate lobe of the liver differs anatomically from the other lobes, as it drains directly into the inferior vena cava (IVC) through veins separate from the main hepatic veins [5]. The right and left branches of the portal vein as well as both branches of the hepatic arteries may supply the caudate lobe. Likely due to this complex blood supply, the caudate lobe usually is spared from metastatic disease to the liver and often hypertrophies to compensate for the loss of normal liver parenchyma.

Studies have shown that malignancies other than NET metastasize to the caudate lobe between 4% and 8% of the time [6-8]. A single institution experience with 150 patients showed that the most common indication to undergo caudate hepatectomy was for metastatic colorectal cancer followed by primary hepatic malignancies - cholangiocarcinoma and hepatocellular carcinoma. [6].

Despite the noted anatomical differences, neuroendocrine tumors commonly metastasize to the caudate lobe [9]. The significance of these metastases is unknown, as is the impact on outcomes and response to regional therapy. We reviewed our institution’s experience with TACE for these lesions to determine the effect of caudate lesions as well as their response to TACE.

## Methods

Between 1992 and 2008, TACE was undertaken in 198 patients with neuroendocrine tumor metastases to the liver. Although pre-TACE imaging was undertaken in all patients, a subset of 98 patients had pre-TACE liver imaging (MRI or CT scan) performed in our system and available for this retrospective review. Two independent surgical oncologists (MB and CS) reviewed the patients’ pre-TACE imaging studies to identify those with caudate lobe metastases (CLM). Two groups were identified: 45 patients who presented with evidence of CLM and 41 with no caudate lobe metastases (NCLM). A third sub-group (*n* = 12), those who developed caudate lesions during the course of therapy, was excluded. Demographics, clinicopathologic characteristics, procedure-related complications, symptomatic, radiographic, and biochemical response to TACE as well as outcomes were compared between these two groups. The Ohio State University Institutional Review Board approved this project.

### Transarterial chemoembolization procedure

TACE was considered for inability to control typical carcinoid symptoms with octreotide therapy, liver tumor progression, or liver tumor burden that would threaten liver function if any progression occurred. Eligibility criteria for TACE included a tissue diagnosis of well or moderately differentiated neuroendocrine tumor, serum bilirubin <3 mg/dL, serum creatinine of <2 mg/dL, normal coagulation profile, and platelet count >100,000/mL.

Although our institution initially preferred whole-liver TACE, in the more recent cohort of patients, a sequential lobar approach was adapted [10]. For staged TACE, the liver lobe with greater tumor burden was treated first. Any subsequent TACE treatments were timed according to the patient’s symptoms, response, and how well the initial procedure was tolerated. All treatment decisions were made in a multidisciplinary setting including the treating surgical, medical, and interventional oncologists.

On the morning of the procedure, the patients were first placed on a continuous intravenous octreotide infusion, which continued until the day after the TACE. Patients with carcinoid syndrome were also maintained on octreotide in the outpatient setting before and after TACE. Prophylactic broad-spectrum antibiotics were routinely administered. All procedures were undertaken in the angiography suite under conscious sedation. A diagnostic visceral angiogram via the femoral artery was done to review the anatomy and verify portal vein patency. After cannulating the hepatic artery of interest, the chemoembolic agents were injected (doxorubicin 30 mg, mitomycin 30 mg, cisplatinum 50 mg, ioxaglate sodium, and ethiodized oil 37%). Embolic particles were then infused until arterial stasis was achieved. Patients were then monitored in the hospital until able to tolerate oral hydration and narcotics.

### Response to TACE assessment

Radiographic, symptom, and biochemical responses to TACE were assessed at 3- to 6-month intervals. CT or MRI was used to assess radiographic response to TACE. Radiographic response was determined by the attending radiologist’s overall impression according to the Response Evaluation Criteria in Solid Tumors (RECIST) criteria and loss of tumor enhancement consistent with necrosis [11].

Subjective symptom response was assessed by review of clinic charts based on patient and physician assessment. Worsening of symptoms as described by the patient or the need for higher doses of octreotide to control symptoms were considered progression of disease. Stable disease was defined as no change in symptoms after TACE. Improvement in symptoms was considered partial response. Complete response was defined as the complete resolution of symptoms.

The biochemical response to TACE was assessed by determination of plasma pancreastatin levels [12]. Pre-procedure pancreastatin levels were used as baseline. Post-TACE pancreastatin levels that decreased by 20% or more were considered a partial response, while normalization of a previously elevated pancreastatin was considered a complete response. Any increase of serum pancreastatin level after reaching a nadir was considered progression of disease.

### Statistics

Overall survival curves were constructed using the Kaplan-Meier method, and comparisons were made using log-rank analysis. Overall survival was determined from the time of the first TACE until death from any cause as determined by medical records or social security death index (ssdi.rootsweb.ancestry.com). Of note, all deaths in this cohort were attributed to primary disease. Independent predictors of survival were determined by multivariate analysis by using the Cox proportional hazards model. Categorical data were compared using chi-square or Fisher’s exact test, and continuous data were compared by Mann-Whitney *U* or Student’s *t* test, where appropriate. All *P* values were derived from two-tailed tests. All statistical analyses were completed using SPSS v 17.0 software (SPSS, Inc., Chicago, IL, USA).

## Results

Patients with CLM and those with NCLM had no significant differences in terms of age, gender, and comorbidities (Table 1). Commensurate with their age, significant comorbidities - including coronary artery disease, hypertension, and diabetes - were present in 31% of the patients in the CLM group and in 44% in the NCLM group (*P* = NS). Patients in both groups were as likely to present with carcinoid syndrome at the time of TACE. The primary tumor was more likely to have been removed in the NCLM group (66%) than in patients with CLM (38%) (*P* < 0.03). Both groups were similar in percentage of patients with a pancreatic primary tumor, percentage of patients with a non-functional tumor at presentation, and tumor grade (Table 1).

**Table 1:** **Demographics and clinicopathologic characteristics of 86 patients**

**Variables**	**Caudate lobe metastases (** ***n*** **= 45)**	**Number of caudate lobe metastases (** ***n*** **= 41)**	***P*** **value**
Mean age (range)	54.8 (29 to 85)	53.5 (28 to 75)	0.85
Gender M:F	17:28	20:21	0.29
Comorbidities	14 (31%)	18 (43.9%)	0.45
Carcinoid syndrome pre-TACE	38 (84.4%)	35 (85.4%)	.92
Primary resected	17 (38%)	27 (65.8%)	<0.03
Pancreas primary	10 (22%)	10 (24.4%)	0.096
Tumor non-functional at presentation	30 (65.9%)	20 (48.9%)	0.085
Tumor grade			0.795
1	36 (80%)	33 (80.5%)	
2	4 (8.9%)	4 (9.8%)	
3	2 (4.4%)	2 (4.9%)	
Median pre-TACE pancreastatin level pg/ml (range)	12,856 (84 to 56,200)	5,985 (104 to 46,400)	<0.03
Median number of liver segments with metastases (range)	8 (5 to 8)	5 (2 to 8)	<0.05
Median (range) percentage of metastatic burden to liver	71.4% (30 to 95)	48.7% (5 to 95)	<0.01
Presence of extrahepatic disease	32 (71%)	22 (54%)	0.24
Presence of lymph node metastases	9 (22%)	7 (15.6%)	0.489

Hepatic tumor burden was greater in patients with caudate lobe metastasis in terms of number of liver segments involved, estimated proportion of liver involvement, and pre-TACE pancreastatin levels (Table 1). At the time of TACE, all patients had radiographic evidence of bilobar hepatic disease. Additionally, 62% of the entire cohort had evidence of extrahepatic disease, which was statistically similar between groups. Pre-TACE pancreastatin levels were abnormally elevated in 80 patients (normal pancreastatin level <135 pg/ml). A similar percentage of patients in each group had an elevated pancreastatin level (87% *vs*. 85%).

A total of 112 TACE procedures were undertaken in 86 patients, with a mean of 1.2 (range 1 to 2) per patient. Patients with CLM were less likely to undergo whole-liver TACE (26.7% *vs*. 56.1% of patients in the NCLM cohort, *P* = 0.005). Twenty-two patients underwent planned staged TACE procedures with only one lobe of the liver being treated during each session. Ten post-procedural complications occurred in the total cohort, resulting in two deaths, one in each group. Both deaths were a result of liver failure leading to multisystem organ failure in patients with heavy hepatic tumor burden (that is, >75%). Complication rates were similar between groups. Atrial fibrillation with rapid ventricular response requiring medical therapy was the most common complication, occurring in four patients. Intrahepatic abscess formation occurred in two patients that required percutaneous drainage. Two patients developed severe hypertension requiring continuous monitoring, and one of these suffered a subarachnoid hemorrhage from a ruptured aneurysm that required neurosurgical intervention. Right upper quadrant pain, fatigue, transient rise in liver transaminases, and fevers after TACE were not considered complications as they occur commonly after this procedure. The mean length of stay was similar between groups (Table 2).

**Table 2:** **Outcomes following transarterial chemoembolization for metastatic neuroendocrine tumors**

**Variables**	**Caudate lobe metastases (** ***n*** **= 45)**	**Number of caudate lobe metastases (** ***n*** **= 41)**	***P*** **value**
Median length of stay in days (range)	5.9 (1 to 27)	4.7 (1 to 17)	0.23
Radiographic response^a^	37/45 (82.2%)	34/41 (82.9%)	.84
Mean duration of radiographic response in months (range)	14.5 (3 to 61)	12.9 (2 to 71)	0.41
Symptom response^b^	29/45 (64.4%)	23/41 (56.1%)	.51
Median duration of symptomatic response in weeks (range)	12 (2 to 43)	12.5 (2 to 73)	0.30
Biochemical response^c^	40/41 (97.6%)	32/36 (88.9%)	0.18^*^
Median duration of biochemical response in weeks (range)	13 (1.5 to 53)	17.4 (2.1 to 37.8)	0.91

Percentages are calculated based upon the number of patients with complete data. ^a^Denominators vary based upon availability of post-TACE data; ^b^denominators based upon number of patients with carcinoid syndrome prior to TACE; ^c^denominators vary based upon availability of pancreastatin levels.*Fisher’s exact probability test.

Radiographic, symptomatic, and biochemical response to TACE was similar between groups as was duration of response (Table 2). Thirty-eight patients in the CLM group had post-TACE CT scans available for comparison. In this group, 79% demonstrated stable disease or partial response to TACE with eight showing progression of disease. In the NCLM group, 39 had post-procedure radiographic imaging. Similar to those with CLM, 82% had stabilization or regression of disease after TACE, while seven progressed. In patients with CLM, only seven caudate lesions (15.5%) responded to TACE. There was no significant difference in the control of symptoms in either group following TACE. Only one patient reported worsening of symptoms following TACE. CT scan noted this patient, in the NCLM group, to have progression of disease. As assessed by changes in pancreastatin level, there was no difference in the likelihood or duration of biochemical response between groups following TACE.

**Figure 1:**
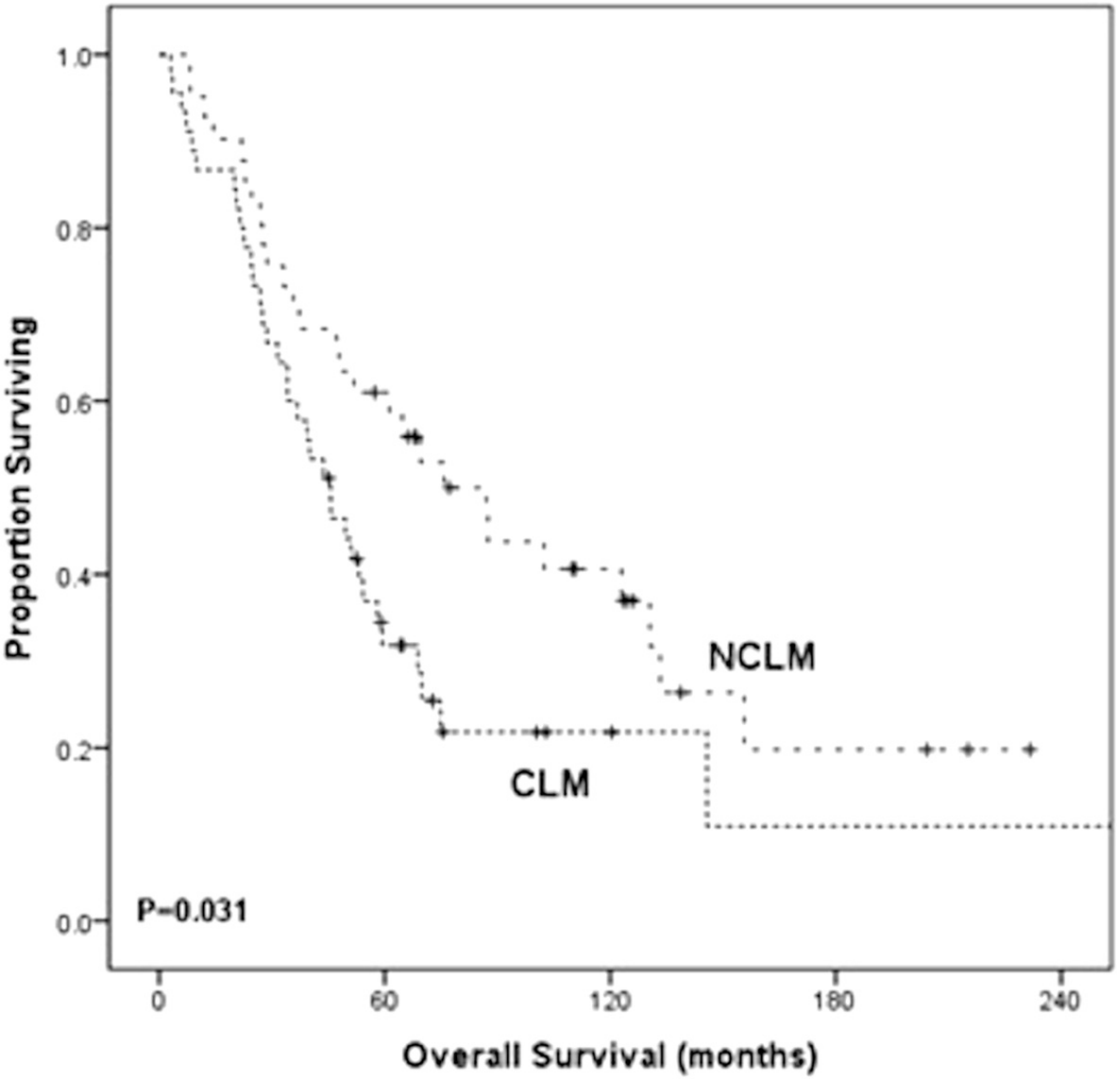
Overall survival curve in patients with (CLM) and with no (NCLM) caudate lobe metastases from neuroendocrine tumors. Kaplan-Meier curves with log-rank test were performed to compare survival between the cohort of patients with caudate liver metastases (CLM) *versus* those without such lesions (NCLM). Patients with liver metastases, but sparing of the caudate lobe, had significantly improved survival compared to those with caudate lobe lesions (87.1 *vs*. 45.6 months, *P* = 0.031).

Overall survival for the NCLM group was significantly longer than patients with CLM (Figure 1). Those in the NCLM cohort had a median overall survival of 87.1 months with 59% alive at 5 years *versus* 45.6 months and 36% at 5 years in the CLM cohort. When comparing the presence of CLM with other factors related to patient survival, including demographics, details of primary disease, and extent of metastatic disease (Table 3), the presence of CLM was not an independent predictor of survival. In patients with caudate lesions whose disease elsewhere in the liver responded to TACE, failure of response in the caudate lobe yielded similar overall median survivals to those who had responsive caudate lesions (29 *vs*. 39 months; *P* = NS).

**Table 3:** **Predictors of overall survival after transarterial chemoembolization in patients with liver metastases from neuroendocrine tumors**

**Patient factor**	**Univariate analysis**	**Multivariate analysis**
	**(** ***P*** **value)**	**(** ***P*** **value)**
Presence of caudate metastases	0.031	
Age over 50	<0.001	0.004
Gender	0.329	
Race	0.170	
Comorbidities	0.363	
Number of liver segments involved	0.001	
Percent of liver involved	<0.001	0.003
Primary resected	<0.001	<0.001
Pancreatic primary	<0.001	
Tumor grade	0.422	
Presence of lymph node metastases	0.589	
Functional tumor	0.047	
Presence of extrahepatic disease	0.004	

## Discussion

Between 50% and 75% of patients with neuroendocrine tumors develop metastatic lesions in the liver, and these lesions are rarely amenable to curative resection. Morbidity in these patients arises from liver symptomatology resulting in a significant decrease in quality of life [13]. Liver-directed therapies such as TACE have been successful at prolongation of life and improving quality albeit in the name of palliation [14]. We identified a subgroup in which metastases also occurred in the caudate lobe, finding that these lesions are often harbingers for aggressive pathology as patients with CLM have reduced median overall survival. Of note, when compared to other patient factors, the presence of CLM was not a significant predictor of survival by multivariate analysis. To our knowledge, no literature to date has described the impact of metastatic neuroendocrine tumor to the caudate lobe.

At our institution, caudate lobe metastases were identified in 52% of patients who presented for TACE. While the incidence of CLM in NET has not been described previously, it is clearly more frequent than other histologic tumor types where a much lower incidence of CLM has been reported, ranging from only 4% to 8% of patients [6-8]. A single institution’s experience with 150 patients showed that the most common indication to undergo caudate hepatectomy was for metastatic colorectal cancer, followed by cholangiocarcinoma and hepatocellular carcinoma [6]. The survival and oncological outcomes after isolated caudate lobe resection or in combination with a larger hepatic resection for metastasis from colorectal cancer are similar to those seen in patients without caudate lobe metastases. Long-term survival is comparable to metastases elsewhere in the liver; provided principles of hepatic surgery are followed [8].

In our study, we investigated all patients who underwent TACE and had caudate lobe metastases. Patients were included in the CLM group if they had radiographic evidence (CT or MRI) of caudate lobe involvement regardless of the size of the lesion. Patients with CLM tended to have a greater tumor burden and were less likely to have had resection of the primary when compared to NCLM patients. No significant differences existed when comparing symptomatology, specifically with respect to carcinoid-related complaints. This is important to recognize because the presence of carcinoid syndrome has been shown to be predictive of better outcome after liver-directed therapies [15-19].

TACE for metastatic neuroendocrine tumor is a safe procedure but still associated with complications. In our study, patients undergoing this procedure had a 10% complication rate with a 2% mortality rate. Post-TACE events such as the classic TACE syndrome (right upper quadrant pain, nausea, vomiting, fatigue, transient rise in liver transaminases, and fevers) are common and were not considered complications [19,20]. The two peri-procedural deaths in our study were related to acute liver failure leading to multisystem organ failure. Each of these patients had undergone whole liver chemoembolization early in our experience and had large tumor burden. Since these events, our practice has moved to using staged lobar embolizations in order to reduce liver-related complications.

The majority of patients with carcinoid syndrome experienced symptom improvement after TACE regardless of caudate lobe involvement. This was assessed at each clinic by way of a complete history and physical examination. In addition, any increase in octreotide dosage or report of new or worsening symptoms, no matter how minor, was interpreted as a sign of progressive disease. This most likely underestimates the real durability of symptom response after TACE, but it is the most objective criteria to assess progression of disease in this retrospective study.

The biochemical response to TACE was evaluated by the use of pancreastatin as a marker. Pancreastatin is a split product of chromogranin A and has been shown to be a sensitive marker in neuroendocrine tumors [21,22]. We have shown that a greater than 20% decrease in pancreastatin correlates with improved outcome after TACE, including improved survival [12]. Using the same parameters, we found similar proportion of patients achieving this threshold in both groups indicating that overall reduction in viable tumor burden was accomplished regardless of the response in the caudate.

Radiographic response was assessed by comparison of pre-TACE CT scans and/or MRIs to post-TACE imaging. RECIST is not well suited for regional therapy because RECIST relies on clear measurement of target lesions and assumes that changes in these target lesions are reflective of changes in all lesions within the entire organ. Although this method is beneficial when comparing efficacy of systemic therapies, it has inherent flaws when comparing embolic or particle therapy where tumors may receive varying amounts of drug. It is unclear if RECIST criteria should be applied to unilobar TACE, where tumors often regress on the treated side but progress in the non-treated lobe. Even less clear is how to assess lesions that have developed calcifications or necrosis after treatment with TACE but have remained the same diameter. For these reasons, we decided to utilize the radiologist’s interpretation, which usually incorporated RECIST, to simply classify tumors as regressive, stable, or progressive [11,23]. Radiographic stabilization or improvement of disease was seen in 82% of patients. However, on review of the post-TACE imaging, response specifically in the caudate was uncommon (15.5%) irrespective of the response elsewhere in the liver. This lack of response could be explained by alterations in arterial anatomy of the caudate lobe compared to the other lobes of the liver.

There are several limitations to the current study. Firstly, it is a retrospective review and as such is susceptible to biases inherent to such a study. We reviewed data at our institution alone. We use TACE as our regional therapy of choice for NET metastases to the liver, using similar techniques for chemoembolization each time. Although it is possible differing administration of chemoembolic materials may impact outcomes, our technique is the standard for this therapy. It is also possible that other regional therapies, such as bland embolization or radioembolization, may vary in the impact on caudate lesions. Such comparisons would be beyond the scope of this study. Although all deaths were attributed to disease, our database did not parse out exact cause in order to differentiate those who succumbed to liver failure *versus* other causes. Although several recent studies [24-26] have shown the importance of extent of primary tumor and presence of peritoneal carcinomatosis in determining outcome in small bowel NET, our database did not collect this data and, as such, could not be used to compare between groups. In sum, this study provides insight into the impact of TACE, specifically, in the treatment of caudate NET metastases.

## Conclusions

Although patients with or without caudate metastases have similar radiographic, symptomatic, and biochemical responses to TACE, the lesions in the caudate lobe themselves respond poorly to TACE compared to lesions in other lobes of the liver. Additionally, overall survival after TACE was negatively impacted by the presence of a caudate lesion. It is unlikely that the mere presence of CLM affects survival given that when disease elsewhere in the liver responded to TACE, failure of response in the caudate lobe yielded similar overall survival to those who had responsive caudate lesions. Instead, CLM may reflect greater tumor burden. We show that these lesions do not respond well to TACE in spite of typically good response in the remainder of the liver. Given the long duration of patient survival, caudate lesions may become quite large, symptomatic, or encroach upon portal structures. Given the inability of TACE to control growth, these lesions may best be treated with resection in order to palliate these effects.

## Abbreviations

CLM: caudate lobe metastases
NCLM: no caudate lobe metastases
RECIST: Response Evaluation Criteria in Solid Tumors
TACE: transarterial chemoembolization
